# A dataset of the mid-brunhes period at site MD05-2925, Solomon Sea: Surface-subsurface planktonic foraminifera stable C-O isotope and Mg/Ca ratios

**DOI:** 10.1016/j.dib.2021.107283

**Published:** 2021-08-12

**Authors:** Li Lo, Tzu-Ling Chang, Yu-Chu Su, Chih-Kai Chuang, Chuan-Chou Shen, Horng-Sheng Mii

**Affiliations:** a Department of Geosciences, National Taiwan University, Taipei 10617, Taiwan; b Paleoceanography Laboratory, National Taiwan University, Taipei 10617, Taiwan; c Research Center for Future Earth, National Taiwan University, Taipei 10617, Taiwan; d Taipei First Girls High School, Taipei 10045, Taiwan; e High-Precision Mass Spectrometry and Environment Change Laboratory, National Taiwan University, Taipei 10617, Taiwan; f Department of Earth Sciences, National Taiwan Normal University, Taipei 11677, Taiwan

**Keywords:** Solomon Sea, Planktonic foraminifera, Benthic foraminifera, Oxygen isotope stratigraphy, Carbon isotope, Mg/Ca ratios, Seawater oxygen isotope, Marine Isotope Stage 11

## Abstract

Here we present derived thermal-hydrological variations data during the Marine isotope stages (MISs) 10–12 using surface and subsurface dwelling planktonic foraminiferal geochemical proxies of a sedimentary core of MD05-2925 (9.3^o^S, 151.5^o^E, water depth 1661 m, core depth 1842–2430 cm), Solomon Sea. *Globigerinoides ruber* (*s.s.*, white, 250–300 µm) and *Pulleniatina obliquiloculata* (355–425 µm) tests were hand-picked and cleaned for stable carbon and oxygen isotopes and Mg/Ca analyses. Composite benthic foraminifera tests (>250 µm, *Uvigerina spp.*, and *Bulimina spp.*) are also hand-picked and cleaned for stable oxygen isotope stratigraphy. In total, 235 and 148 measurements for C-O stable isotopes and Mg/Ca ratios for planktonic foraminifera in 2–5 cm resolution for the period from 352.1 to 462.3 ka are presented in this data report, respectively. Age model is established by tuning composite benthic foraminiferal oxygen isotope to global composite benthic foraminifera oxygen isotope stack LR04. Surface and subsurface temperatures and seawater oxygen isotopes (δ^18^O_W_, without ice volume correction) were calculated.

## Specifications Table

**Table:** 

Subject	Oceanography
Specific subject area	Paleoceanography
Type of data	Raw data Table
How data were acquired	Sediment samples were firstly washed and sieved. Foraminiferal tests were hand-picked under microscope, then cleaned for stable oxygen isotope Micromass IRMS and Mg/Ca ratios by SF-ICP-MS (Element 2, Thermo^TM^ Scientific)
Data format	Raw data
Parameters for data collection	Time: 352.1-462.3 ka Place: Solomon Sea, southwestern equatorial Pacific Indexes: benthic foraminiferal oxygen isotope stratigraphy, surface-subsurface planktonic foraminiferal carbon-oxygen isotope, Mg/Ca ratios and inferred seawater temperatures, and temperature and seawater oxygen isotopes.
Description of data collection	This dataset is collected from sediment washing and sieving, foraminiferal tests picking, cleaning for different chemical analyses. MD05-2925 foraminiferal oxygen isotope and Mg/Ca ratios were measured at Department of Earth Sciences, National Taiwan Normal University and Department of Geosciences, National Taiwan University, respectively.
Data source location	Institution: Department of Geosciences, National Taiwan University City/Town/Region: Taipei Country: Taiwan
Data accessibility	DOI: https://doi.org/10.17632/9c2nnpchdh.1[1]
Related research article	Li Lo, Sheng-Pu Chang, Kuo-Yen Wei, Shih-Yu Lee, Chuan-Chou Shen, Tsong-Hua Ou, Yi- Chi Chen, Chih-Kai Chuang, Horng Sheng Mii, George S. Burr, Min-Te Chen, Ying-Hung Tung, Meng-Chieh Tsai. Nonlinear climatic sensitivity to greenhouse gases over past 4 glacial/ interglacial cycles. Scientific Reports 2017, 7, 4626, https://doi.org/10.1038/s41598-017-04031-x

## Value of the Data

• Composite benthic foraminiferal oxygen isotope stratigraphy sets foundation for age correlation across regions. Solomon Sea vertical thermal and hydrological profiles across the MIS 11 provide insight to study Indo Pacific Warm Pool (IPWP) dynamics during the past warm interglacial period.
• This dataset is in particular useful for regional paleoceanographic and paleoclimatological studies, however, it would be important for global compilations or physical simulations as well.
• Composite benthic foraminiferal oxygen isotope data could be used to revise regional oxygen isotope stratigraphy. Surface and subsurface temperature-oxygen isotopes and their gradients could be used to reconstruct the dynamics in the south marginal of IPWP.

## 1 Data Description

The dataset contains four worksheets, including depth, planktonic foraminifera oxygen isotope (δ^18^O_C_), Mg/Ca ratio and inferred temperature, calculated seawater oxygen isotope and benthic foraminifera oxygen isotope data, age control point, and average sedimentation rate. We adopted Anand et al. [2] Mg/Ca-temperature equation to calculate seawater temperature for the selected two planktonic foraminiferal species. To extract seawater oxygen isotope (δ^18^O_W_, without ice volume effect correction) information, we used an equation of T = 16.5 − 4.8 × (δ^18^O_C_ − δ^18^O_W_) [3] and a constant offset of 0.27‰ between VPDB and VSMOW scales. The main contribution of uncertainty of Mg/Ca derived temperatures is mostly from Mg/Ca-temperature estimation and an overall error of ∼1.2 °C is reported by Anand et al. [2].

The most abundant benthic genus in MD05-2925 is *Uvigerina spp.*; however, in several levels, especially during termination periods, we could not find enough *Uvigerina spp.* tests (<2 individuals) for benthic foraminifera oxygen isotope analysis. Previous study from MD05-2925 [4] adapted composite benthic foraminifera oxygen isotope stratigraphy for the past 350-kyr. Here we report the original oxygen isotope records from different species/genus in an excel worksheet. In total, there are four age control points are acquired by tuning to global benthic foraminiferal stack LR04 [5] in the study period and calculated sedimentation rates ranged from 3.1–6.5 cm/kyr.

In this dataset we report depth in sediment core, age (ka), geochemical data (δ^18^O_C_, δ^13^C_C_ and Mg/Ca ratios) and climatic parameters derived from geochemical proxies (temperatures and δ^18^O_W_) in both worksheets for *G. ruber* and *P. obliquiloculata*. Composite benthic foraminifera oxygen without species corrections in depth and age in benthic foraminiferal worksheet. Finally, age control points and average sedimentation rate are reported in respectively worksheet.

## 2 Experimental Design, Materials and Methods

### 2.1 Experimental design

We hand-picked well-preserved, unbroken and without clear dark spots benthic and planktonic foraminifera tests. Specific cleaning procedures for stable isotope and Mg/Ca were then conducted for subsamples before geochemical analyses [4]. Off-line data reduction and standard calibrations were also introduced after analyses by using standard bracket method. Planktonic foraminiferal Mg/Ca ratios are then converted to temperature and seawater oxygen isotope values then calculated based on the respective species δ^18^O_C_ and corresponding Mg/Ca temperature values [2,3].

### 2.2 Materials

The marine sediment core, MD05-2925, 2843 cm in length, and recovered during the IMAGES XIII-PECTEN (Past Equatorial Climate: Tracking El Niño) cruise on board the R.V. Marion Dufresne of the French Polar Institute (IPEV) in 2005. Sediment samples are stored in the Taiwan Ocean Research Institute (TORI). Subsamples and the remnant sediment samples are achieved in the Department of Geosciences, National Taiwan University.

### 2.3 Methods

Typically, 30–50 planktonic foraminifera *G. ruber* and *P. obliquiloculata* tests of each sediment subsample were hand-picked under the microscope and prepared for geochemical analyses. For Mg/Ca analyses, 20–30 foraminiferal tests were crushed within teflon vials with a teflon bar and left in a 1.5 mL Teflon vial. The test fragments were cleaned with the following reagents: (1) ethanol, (2) H_2_O_2_ (0.45 mL, 1%), (3) NH_4_Cl (0.45 mL, 1.0 N), (4) NH_2_OH (0.45 mL, 0.01 N), and (5) dilute nitric acid (1 mL, 0.005 N. [5]. A sector field inductive coupled plasma mass spectrometer (SF-ICP-MS), Element 2, housed at the High-Precision Spectrometry and Environment Change Laboratory (HISPEC), Department of Geosciences, National Taiwan University, was used to determine metal/Ca ratios. The long-term performance of 1σ reproducibility of Mg/Ca analyses is ± 0.21% [6].

For oxygen stable isotope analysis, 7–10 planktonic and 2–4 benthic foraminiferal tests were immersed in methanol, ultrasonicated for 10 s, and then rinsed with deionized water 5 times. Samples were immersed afterward in sodium hypochlorite (NaOCl) for 24-hr, and then analyzed with an isotopic ratio mass spectrometer (IRMS), Micromass IsoPrime, at the National Taiwan Normal University. The long-term 1σ precision of this instrument is better than ±0.05 ‰ (*N* = 701, [4] and data are reported with respect to the Vienna Pee Dee Belemnite (VPDB) standard through the calibration of NBS-19 (National Bureau Standards; δ^18^O = −2.20 ‰).

## Ethics Statement

N/A.

## CRediT Author Statement

**Li Lo:** requested samples and designed this study; **Tzu-Ling Chang** and **Yu-Chu Su:** picked planktonic foraminifera, sample preparations and measured Mg/Ca ratios; **Chih-Kai Chuang, Chuan-Chou Shen, Horng-Sheng Mii** and **Li Lo:** helped on instrument operations, oxygen stable isotope analyses and all authors contributed manuscript preparation and improvement.

## Declaration of Competing Interest

The authors declare that they have no known competing financial interests or personal relationships which have or could be perceived to have influenced the work reported in this article.

## Appendix Supplementary materials

Image, application 1
